# Efficacy and feasibility of aerobic exercise interventions as an adjunctive treatment for patients with schizophrenia: a meta-Analysis

**DOI:** 10.1038/s41537-023-00426-0

**Published:** 2024-01-02

**Authors:** Jing Guo, Keju Liu, Yundan Liao, Ying Qin, Weihua Yue

**Affiliations:** 1 Peking University Sixth Hospital, Peking University Institute of Mental Health, Beijing, 100191 China; 2 National Clinical Research Center for Mental Disorders (Peking University Sixth Hospital), Beijing, 100191 China; 3 NHC Key Laboratory of Mental Health (Peking University), Beijing, 100191 China; 4 Department of Psychology, Medical Humanities Research Center, Binzhou Medical University, Yantai, 264003 China; 5 Peking University Shenzhen Hospital, Shenzhen, 518036 China; 6 The Second People’s Hospital of Guizhou Province, Guiyang, Guizhou China; 7 PKU-IDG/McGovern Institute for Brain Research, Peking University, Beijing, 100871 China; 8 Chinese Institute for Brain Research, Beijing, 102206 China

**Keywords:** Schizophrenia, Psychosis

## Abstract

Schizophrenia is a chronic mental disorder primarily treated with antipsychotics, which have limited efficacy for negative symptoms. This study aims to evaluate the effectiveness and feasibility of exercise interventions as adjuncts to pharmacotherapy through a meta-analysis, providing valuable insights for rational intervention design. Four databases were searched, and randomized controlled trials with no language restrictions published up to March 27, 2023, were included. The primary outcome indicator was the Positive and Negative Symptom Scale (PANSS) total score along with its three sub-scales. Secondary outcomes included the Scale for Assessment of Negative Symptoms (SANS) and Body Mass Index (BMI), which were used to assess the efficacy of aerobic exercise interventions in patients with schizophrenia. Subgroup analyses were conducted to explore the impact of intervention duration and total weekly exercise time, while treatment feasibility was assessed through adherence rates. A total of 17 publications involving 973 patients with schizophrenia were deemed eligible and included in the analysis. Compared to other forms of adjunctive interventions, the network meta-analysis of 12 PANSS-based studies revealed that adjunctive aerobic exercise interventions were the most effective in reducing overall PANSS scores in patients with schizophrenia, with statistically significant pooled results (MD = −4.84, 95% CI: −5.72, −3.96). Both the PANSS negative symptom subscale (MD = −2.11, 95% CI: −3.26, −0.95) and SANS (MD = −9.11, 95% CI: −11.94, −6.27) indicated that adjunctive aerobic exercise interventions effectively alleviate negative symptoms. Subgroup meta-analysis indicated that 2-3 month interventions involving 100–220 min of exercise per week were the most effective. Additionally, adherence to the adjunctive aerobic exercise regimen was found to be comparable to that of conventional treatment alone. Aerobic exercise interventions, as adjunctive therapy, are an effective measure for reducing PANSS scores in patients with schizophrenia, contributing to the alleviation of both the positive and negative symptoms, and patients demonstrated strong adherence to aerobic exercise.

## Introduction

Schizophrenia is a complex and chronic mental illness. The Diagnostic and Statistical Manual of Mental Disorders (DSM) and the International Statistical Classification of Diseases and Related Health Problems (ICD) serve as operational criteria for diagnosis^1^. The primary manifestations include positive symptoms such as delusions and hallucinations, negative symptoms like social withdrawal and emotional flatness, and a wide range of cognitive dysfunctions^2–4^^.^ These symptoms form the basis for the quantitative assessment of both the illness’s severity and the effectiveness of treatments^5^. Commonly used clinical assessment scales include the Positive and Negative Symptom Scale (PANSS)^6^, the Scale for Assessment of Negative Symptoms (SANS)^7^, the Scale for Assessment of Positive Symptoms (SAPS)^8^, and the Brief Psychiatric Rating Scale (BPRS)^9^.

Current antipsychotic medications effectively treat positive symptoms, such as agitation and aggression, and also provide some relief for suicidal tendencies. However, these medications have shown limited effectiveness in improving negative and cognitive symptoms^10,11^. Improving negative and cognitive symptoms is crucial for the overall recovery of individuals with schizophrenia and their reintegration into normal life^12^. Therefore, there is an urgent need for more effective interventions.

In recent years, numerous clinical trials have explored the efficacy of non-drug treatments by comparing them to drug therapy alone and in combination therapies that include both drug and non-drug treatments. Common non-drug treatments include psychosocial interventions, such as cognitive behavioral therapy, supportive psychological intervention, and social skills training; cognitive remediation therapy; and exercise interventions. These treatments have been shown to be effective in alleviating the negative symptoms of schizophrenia^11,13–15^. Among these non-drug treatments, psychosocial interventions and cognitive remediation therapies require the involvement of professional psychotherapists. These interventions are often time-consuming and financially burdensome^16^. Given the current shortage of psychotherapists and low treatment adherence among patients with schizophrenia, the widespread adoption of these two non-drug treatment methods remains challenging^17^. In contrast, exercise interventions offer the benefits of ease of implementation, broad applicability, and lower costs, making them a more feasible option for widespread adoption^18–20^. Additionally, research indicates that pharmacological treatments for schizophrenia often focus on blocking dopamine D_2_ receptors, which may lead to exercise-related adverse reactions. Notably, exercise interventions have been shown to offer some preventive effects against these adverse reactions^21^. Consequently, exercise interventions may be the most suitable non-pharmacological treatment option for widespread clinical adoption and dissemination.

Exercise interventions can be categorized into aerobic exercise (AE), anaerobic exercise, and high-intensity interval training (HIIT). Among these types of exercise, anaerobic exercise involves greater physical intensity and demands a higher level of physical fitness, making it less suitable for the patient population^22,23^. Existing studies predominantly consist of randomized controlled trials (RCTs) that compare conventional therapy with adjunctive exercise interventions to investigate the therapeutic outcomes. However, there is a lack of meta-an alysis in the context of schizophrenia that explores the types of exercise and intervention settings most appropriate for these patients.

Therefore, this study focuses on exercise interventions for patients with schizophrenia. It employs a network meta-analysis to compare the outcomes of conventional therapy (Treatment as Usual, TAU), conventional therapy with adjunctive AE, conventional therapy with adjunctive aerobic exercise and psychotherapy (AP), and conventional therapy with adjunctive unspecified aerobic exercise (NAE). Concurrently, the meta-analysis was performed to examine the specific effects of aerobic exercise intervention using the PANSS, SANS, and Body Mass Index (BMI) as outcome indicators. Subgroup analyses also considered the length of the exercise intervention and total weekly exercise hours. The identification of a more rational design for exercise intervention is expected to improve recovery outcomes for patients with schizophrenia.

## Methods

We followed the Preferred Reporting Items for Systematic Reviews and Meta-Analyses (PRISMA) guidelines for reporting this meta-analytic review^24,25^. A study protocol was submitted to PROSPERO (International Prospective Register of Systematic Reviews) with the registration number CRD42023415855 prior to conducting the final analysis for this review.

### Literature search strategies

We conducted a search of the following four electronic databases for literature up to 27 March 2023: PubMed, Embase, Web of Science, and Cochrane Library. The search consisted of the following terms as Medical Subject Headings (MeSH) and keywords appropriate to each database. Specifically, the following search keywords were used: (schizophrenia OR “schizophrenic disorders”) AND (“physical activity” OR exercises OR sports OR aerobic OR training) AND (“randomized controlled trial” OR “clinical trials”). The search terms are adapted to the specific database, using a combination of subject terms such as MeSH (PubMed) and Emtree (EMBASE) and free terms.

### Eligibility criteria

According to the abbreviation PICOS, the selection criteria were as follows: Participants (P): the study included patients diagnosed with schizophrenia, excluding those with major medical conditions such as organic brain diseases, liver or kidney dysfunction, and associated cardiovascular diseases, as well as those with a history of substance use disorders. Interventions (I): the study considered interventions involving any form of exercise. Comparators (C): comparisons with patients with schizophrenia undergoing treatment. Outcomes (O): outcome measures included data from the PANSS, SANS, and Body Mass Index (BMI). Study Design (S): investigating different interventions for patients with schizophrenia were included. Literature that did not meet these criteria was excluded.

### Screening and data extraction

Two authors independently conducted the screening and recording processes without influencing each other’s decisions. They used an information extraction table to collect data from the included literature, capturing details such as author, publication year, diagnostic criteria, experimental group settings, intervention duration, exercise frequency, patient type, and outcome indices. Any disagreements between the two authors were resolved through consensus, facilitated by the corresponding author.

### Quality assessment

The quality assessment was conducted using the Cochrane Risk of Bias Tool 2.0 (ROB 2.0)^26,27^. The evaluation encompassed five domains: bias arising from the randomization process, bias due to deviations from intended interventions, bias due to missing outcome data, bias in measurement of the outcome, and bias in selection of the reported result. Within each domain, multiple signaling questions were addressed. Based on the answers to these signaling questions, each domain was categorized as Low Risk, Some Concerns, or High Risk.

### Data synthesis and analysis

A network meta-analysis of different interventions was conducted using the ‘gemtc’ and ‘rjags’ packages in R 4.1.0 software^28^. Outcome indicators were aggregated using Stata 14.0 software.

In the network meta-analysis, network evidence plots were employed to visualize the relationships between different interventions, and these plots also represented the effect of each direct comparison on network effect sizes. A random-effects model was fitted using frequentist methods^29^. Consistency of the model was assessed through a loop-specific approach^30^. Superiority of the interventions was evaluated based on their likelihood of being the best choice, determined by MeanRank and Surface Under the Cumulative Ranking (SUCRA) values^31^.

*I*^*2*^ statistic and *Q*-test were used to evaluate statistical heterogeneity. *I*^*2*^ < 50% and *P* > 0.1 of *Q*-test indicated low heterogeneity. Fixed-effect model was used to merge effect sizes. If significant heterogeneity existed, sensitivity analysis was used. Combined with the included literature, we took PANSS score, SANS score and BMI as the outcome indicators of this meta-analysis and conducted the analysis respectively. If the same scale/measure was used within the same analysis, so mean difference (MD) and 95% confidence interval (95% CI) were used to combine the results. Conversely, standardized mean differences (SMD) were used. Also, subgroup analysis was performed for the duration of the exercise intervention and the number of hours of exercise per week. The results of meta-analysis and subgroup analysis are presented using forest plots. Tests for publication bias were performed using corrected funnel plots and Egger’s test.

All statistical differences were considered significant when the *P* < 0.05.

## Results

### Search results and quality assessment

**Fig. 1:**
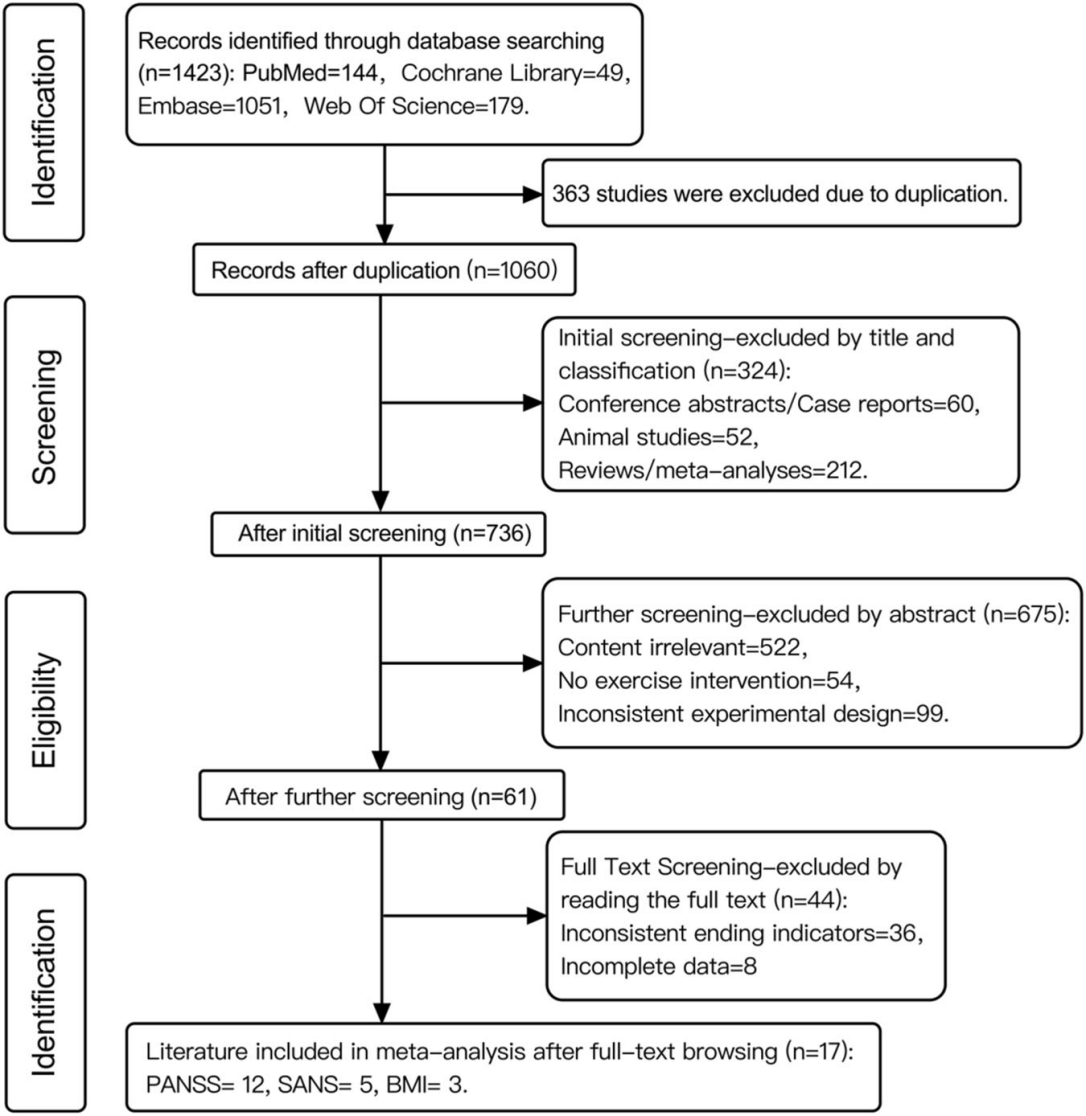
Flow chart of the included studies. The figure represents the amount of literature excluded/included after each step.

A total of 1423 articles were retrieved. Seventeen studies that met the inclusion criteria were selected, comprising 488 patients with schizophrenia who received aerobic exercise intervention and 485 control subjects. The flow chart in Fig. 1 illustrates the literature screening process. Among the seventeen studies included, twelve reported PANSS scores, five reported SANS scores, and three reported BMI values. The patient population included both inpatients and outpatients, and the duration of the exercise interventions ranged from a minimum of one month to a maximum of twelve months. Specific details regarding the lengths of interventions, frequency settings, and other relevant information are presented in Table 1.

**Table 1:** Basic characteristics of the included literature.

NO.	First author (Publication Year)	Diagnostic tools	Experimental / Control group*	Trial duration	Exercise frequency settings	Patient Type	Ending indicators
1	ACIL (2008)^46^	DSM-IV	AE TAU	10 weeks	3 times/week 1 time/day 40 min/time	Inpatient and Outpatient	SANS
2	Rainbow (2012)^47^	DSM-IV	AE TAU	3 months	3 times/week 1 time/day 60 min×2 + 30 min×1	Inpatient	SANS
3	Ikai (2014)^33^	ICD-10	AE TAU	2 months	1 times/week 1 time/day 60 min/time	Outpatient	PANSS
4	Ruiying Kang (2016)^34^	ICD-10	AP TAU	12 months	1 time/two weeks 1 time/day 45 min/time	Outpatient	PANSS
5	Lincoln (2021)^35^	DSM-V	AE NAE	3 months	3 times/week 1 time/day 30-40 min/time	Inpatient	PANSS
6	Siew (2015)^36^	DSM-IV	AE TAU	3 months	3 times/week 1 time/day 30 min/time	Inpatient	PANSS
7	Marzolini (2009)^50^	DSM-IV	AE TAU	3 months	2 times/week 1 time/day 90 min/time	Outpatient	BMI
8	Paikkatt (2015)^37^	DSM-IV	AE TAU	1 months	5 times/week 1 time/day 90 min/time	Inpatient	PANSS
9	Naren (2021)^48^	ICD-10	AE TAU	3 months	7 times/week 1 time/day 60 min/time	Outpatient	SANS
10	Scheewe (2013)^38^	DSM-IV	AE TAU	6 months	2 times/week 1 time/day 60 min/time	Inpatient and Outpatient	PANSS BMI
11	SCHEEWE (2012)^39^	DSM-IV	AE TAU	6 months	2 times/week 1 time/day 60 min/time	Inpatient and Outpatient	PANSS BMI
12	Takeshi (2020)^40^	DSM-V	AE TAU	3 months	2 times/week 1 time/day 60 min/time	/	PANSS SANS
13	Silva (2015)^41^	DSM-IV	NAE TAU	5 months	2 times/week 1 time/day 60 min/time	Inpatient and Outpatient	PANSS
14	Peng-Wei Wang (2018)^42^	DSM-IV	AE TAU	3 months	5 times/week 1 time/day 40 min/time	/	PANSS
15	Dorde (2017)^43^	ICD-10	AE TAU	3 months	4 times/week 1 time/day 45 min/time	Inpatient	PANSS
16	Nami (2022)^44^	DSM-IV	AE TAU	2 months	3 times/week 1 time/day 30 min/time	Inpatient	PANSS
17	Güliz (2020)^49^	DSM-V	AE TAU	3 months	2 times/week 1 time/day 60 min/time	Inpatient	SANS

^*^TAU Treatment as Usual, *AE* Conventional Therapy + Aerobic Exercise Intervention, *AP* Conventional therapy + Aerobic intervention + Psychological intervention, *NAE* Non-single Aerobic Exercise.

**Fig. 2:**
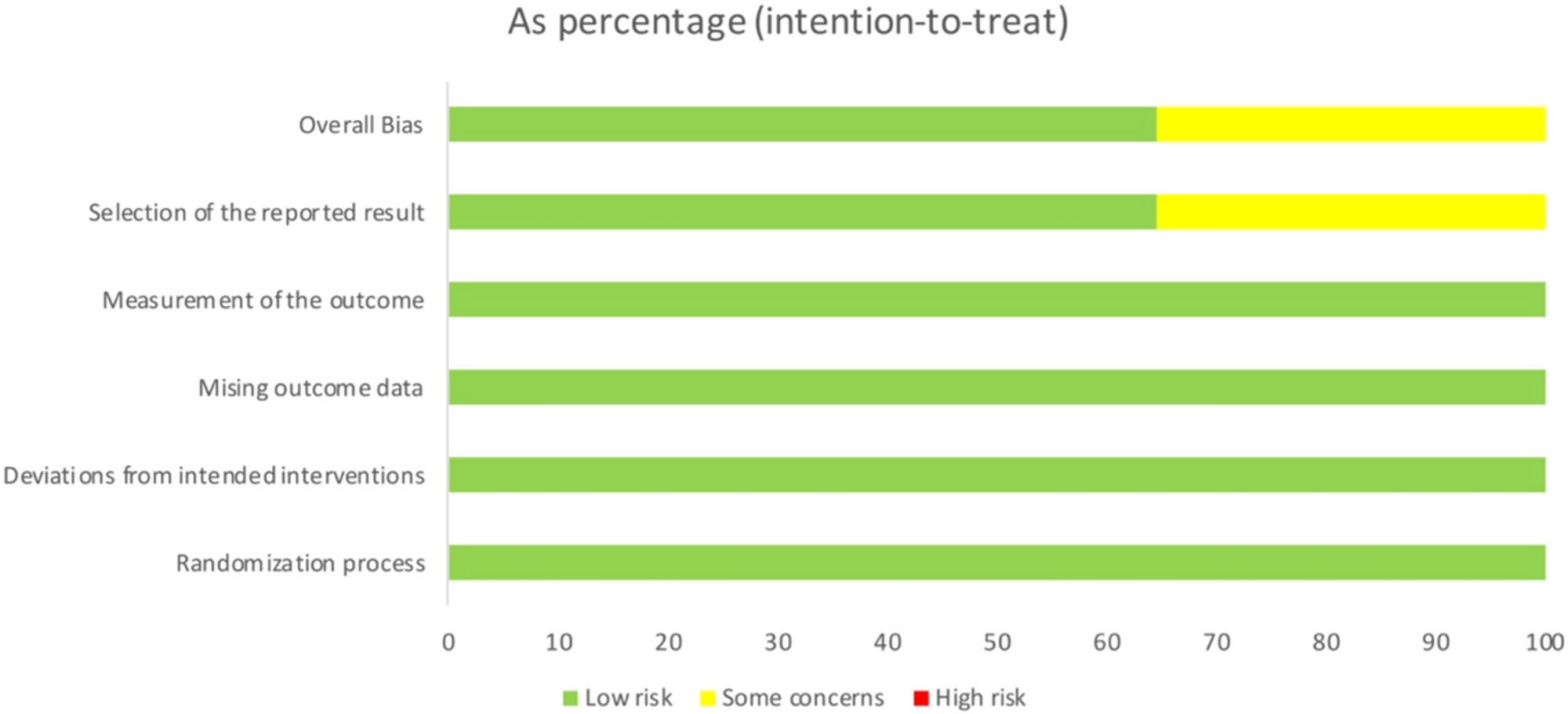
Bias risk analysis of the included literature. Literature quality assessment results showed a good quality and a low bias risk of the included articles.

Quality was assessed according to the risk of bias assessment tool provided by the Cochrane Handbook. All 17 included studies were assessed as ‘Low Risk’ in the domains of Randomization Process, Deviations from Intended Interventions, Missing Outcome Data, and Measurement of the Outcome, indicating no bias risk in these areas. Six studies were evaluated as ‘Some Concerns’ in the domain of Selection of the Reported Result; therefore, the overall quality assessment was rated as ‘Some Concerns’. Specific quality assessments are depicted in Fig. 2.

### Meta-analysis of PANSS

The PANSS is a comprehensive 30-item clinical rating instrument widely used for the assessment of schizophrenia spectrum disorders^32^. It comprises the Positive Scale, the Negative Scale, and the General Psychopathology Scale.

**Fig. 3:**
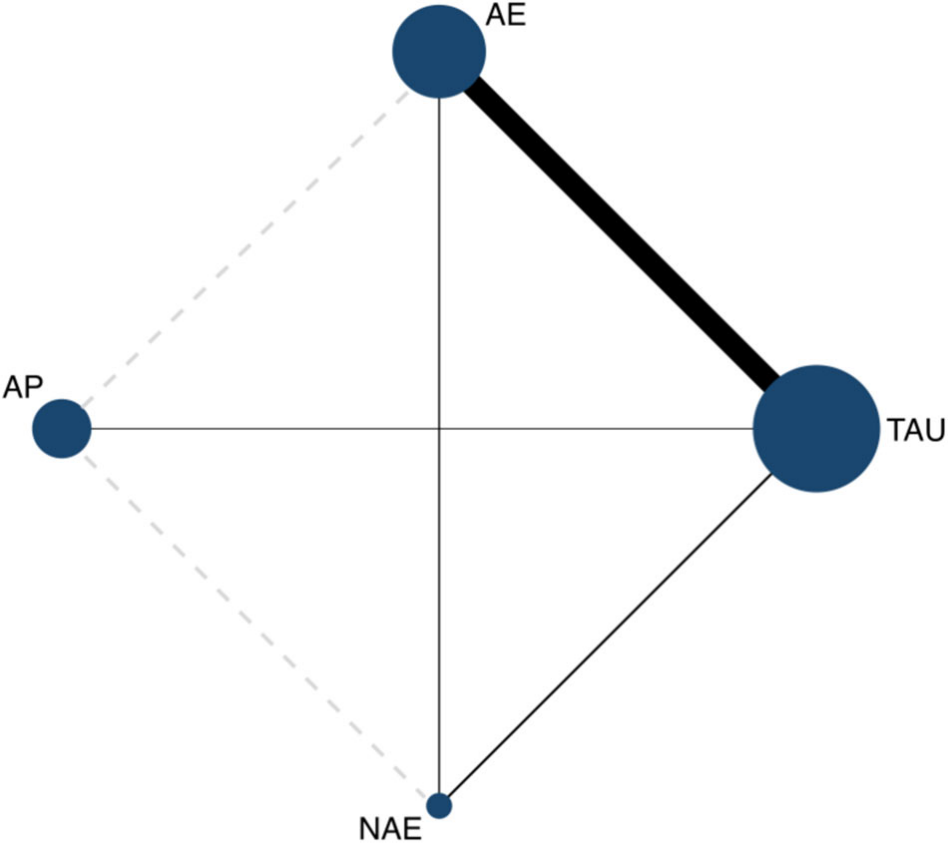
Network evidence plot. *TAU* Treatment As Usual, *AE* Conventional Therapy + Aerobic Exercise Intervention, *AP* Conventional therapy + Aerobic intervention + Psychological intervention, *NAE* Non-single Aerobic Exercise.

Of the 12 studies included^33–44^, there were four direct comparisons and two indirect comparisons. The evidence relationship diagram on PANSS is depicted in Fig. 3. A node in the diagram represents an intervention, and its size represents the number of studies including that intervention. Solid lines between nodes indicate direct comparisons, while dashed lines indicate indirect comparisons. The thickness of the lines represents the amount of literature included for each comparison. The model was fitted based on random effects, with a total of 24 data points and a good fit of 22.83 with a ratio of 0.95. Loop-specific heterogeneity estimates showed an inconsistency coefficient IF = 4.72 for TAU-AE-NAE (95% CI: 0.00–18.24), *P* = 0.49. Additionally, *P*-values in the nodal cut results were greater than 0.05 (Supplementary Fig. 1), indicating that the analysis was consistent and statistically non-significant.

**Fig. 4:**
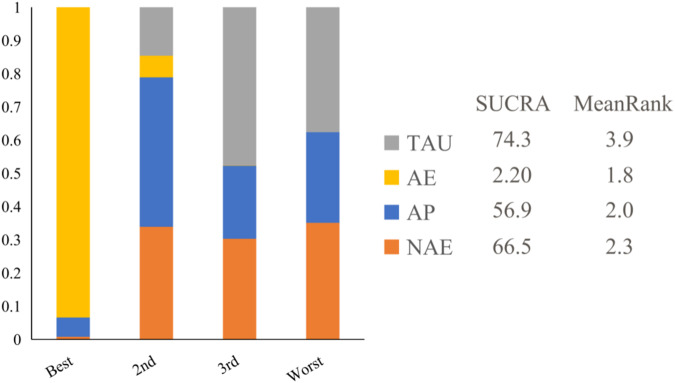
Cumulative ranking of interventions. TAU treatment as usual, AE conventional therapy + aerobic exercise intervention, AP conventional therapy + aerobic intervention +psychological intervention, NAE non-single aerobic exercise, SUCRA surface under the cumulative ranking.

All interventions were ranked based on their SUCRA values, which are expressed as percentages. Lower PANSS scores represent better treatment outcomes; therefore, interventions with lower percentages indicate more effective treatment outcomes. As depicted in Fig. 4, aerobic exercise (AE) appears to be the most effective intervention for reducing PANSS scores in individuals with schizophrenia.

**Fig. 5:**
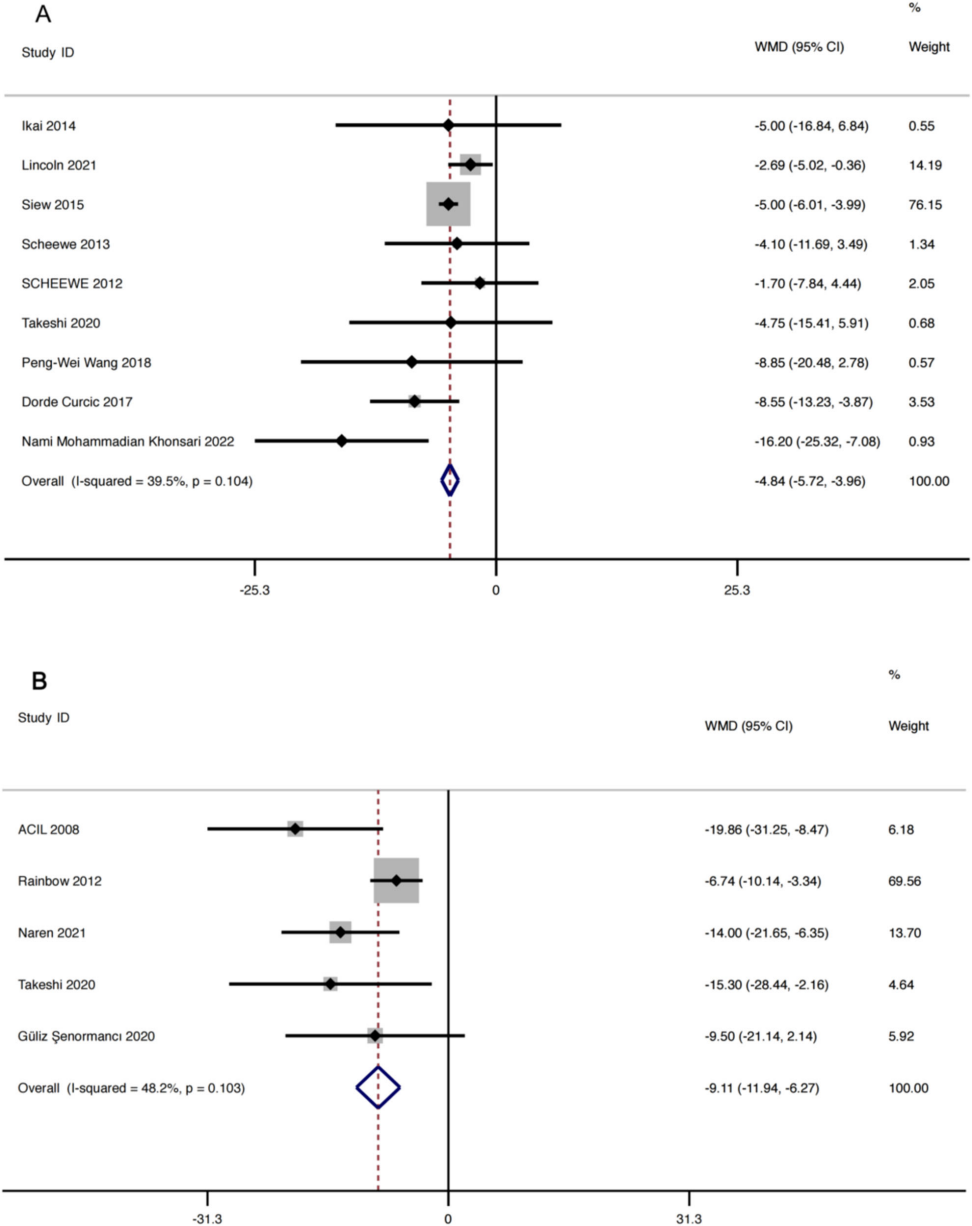
Forest plot. **A** PANSS total score forest plot. **B** SANS total score forest plot.

To further explore the effects of AE on PANSS score reduction, we included ten RCT studies in the meta-analysis. The results indicated a high level of heterogeneity (*I²* = 95.6%, *Q*-test: *P* > 0.1). Sensitivity analysis suggested that the study by Paikkatt in 2015 was the main source of heterogeneity. Upon its exclusion, the heterogeneity fell within a reasonable range (*I²* = 39.5%, *Q*-test: *P* > 0.1). The meta-analysis result yielded MD of −4.84 (95% CI: −5.72, −3.96). Specific results are depicted in Fig. 5A. We then conducted separate subgroup analyses for intervention duration and total weekly exercise duration. To ensure the validity of the subgroup results, data from the Nami 2022 study were excluded following sensitivity analysis. Subgroup analysis of intervention duration showed that AE duration of two to three months was more appropriate for patients with schizophrenia, with MD of −5.81 (95% CI: −8.88, −2.73) (Supplementary Fig. 2A). Subgroup analysis based on total weekly exercise duration indicated that a range of 100 to 220 minutes per week was most effective, yielding MD of −4.82 (95% CI: −5.71, −3.91) (Supplementary Fig. 2B).

Additionally, a meta-analysis of studies reporting scores on PANSS sub-scales was performed. The results showed that aerobic exercise, when combined with conventional treatment, was effective across all three sub-scales (*P* < 0.05), with greater relief of negative symptoms (MD = −2.11, 95% CI: −3.26, −0.95). Subgroup analysis based on the sub-scales revealed that an intervention length of two to three months (MD = −2.05, 95% CI: −3.10, −0.99) was more effective than durations of less than two months.

### Meta-analysis of SANS

SANS serves as a key scale for assessing deficit symptoms in schizophrenia within the framework of schizophrenic disorders^45^. Comprising a total of 25 items, the scale operates on the principle that higher scores denote greater disease severity. Accordingly, lower scores suggest a reduction in negative symptoms.

In this study, SANS scores were reported in five separate investigations^40,46–49^. The heterogeneity among these studies was minimal (*I²* = 48.2%, *Q*-test: *P* > 0.1), justifying the use of fixed effects for data consolidation. Results of the meta-analysis (Fig. 5B) revealed an MD of −9.11 (95% CI: −11.94, −6.27), and these findings were statistically significant (*P* = 0.012). This leads us to suggest that AE is effective in ameliorating the negative symptoms of schizophrenia when compared to TAU.

### Meta-analysis of BMI

A total of three studies were included in the meta-analysis^38,39,50^. Upon testing for heterogeneity and employing fixed-effects modeling (*I²* = 0%, *Q*-test: *P* > 0.1), we found that AE did not yield statistically significant improvements in BMI compared to TAU among patients with schizophrenia (MD = −1.01, 95% CI: −2.42, 0.40; *P* = 0.16).

### Publication bias

**Fig. 6:**
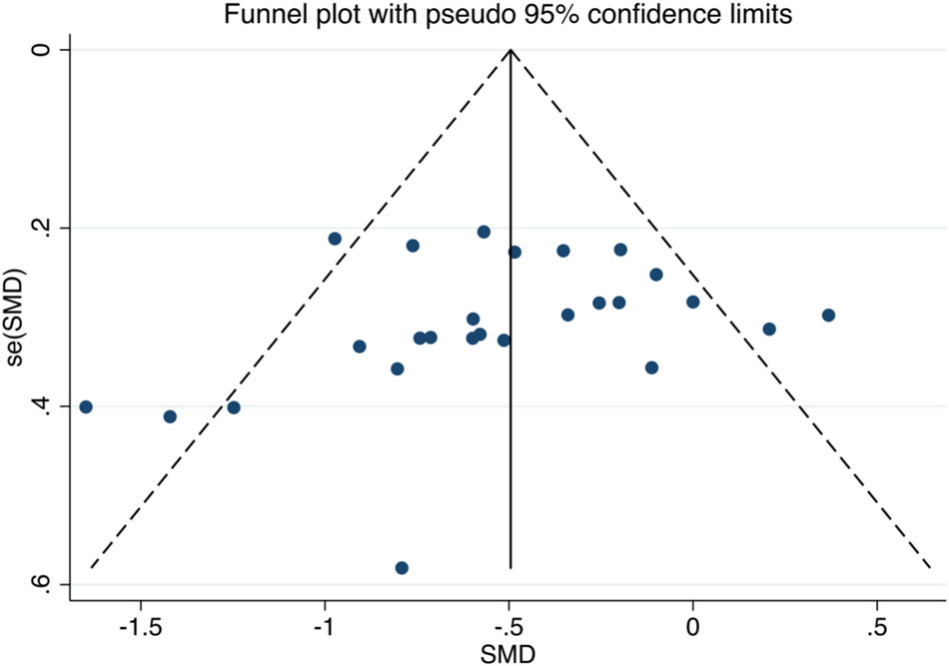
Overall funnel chart. The dotted lines represent 95% confidence intervals, and the dots represent studies.

Initially, we constructed a funnel plot to assess overall publication bias, as illustrated in Fig. 6. The funnel plot appears largely symmetrical, indicating a low risk of overall publication bias for this study. To substantiate this observation, we conducted Egger’s test, which yielded a *P*-value of 0.26, suggesting that no significant publication bias exists in this meta-analysis.

In addition to the overall analysis, Egger’s test was applied to each individual subgroup. The findings corroborate the absence of significant publication bias across all subgroups, as detailed in Table 2.

**Table 2:** Summary of results and publication bias tests.

Scale Categories	I^2^ (%)^a^	MD/SMD^b^	95% CI	Pooled effect size test		Egger’s test- *P*^d^
				Z	*P*^c^	
PANSS total	39.5	−4.84	−5.72, −3.96	10.79	0.002	0.523
Subgroup-Duration of intervention						
Duration <2 months	37.0	−4.64	−5.56, −3.72	9.86	0.001	0.69
2≤ Duration ≤3 months	0.1	−5.81	−8.88, −2.73	3.70	0.001	0.74
Subgroup-Duration of exercise/week						
100≤ Duration ≤220 min	19.6	−4.81	−5.71, −3.91	10.48	0.001	0.71
220mim ≤ Duration	0.1	−2.65	−7.42, 2.12	1.09	0.278	/
PANSS sub-scale						
Positive Scale	0.1	−1.61	−2.15, −1.06	5.79	0.001	0.255
Negative Scale	21.8	−2.11	−3.26, −0.95	3.58	0.001	0.724
General Psychopathology Scale	37.2	−2.08	−2.83, −1.32	5.39	0.001	0.701
Subgroup-Duration of intervention						
Duration < 2 months	0.1	−1.77	−2.22, −1.32	7.75	0.001	0.878
2≤ Duration≤ 3 months	20.0	−2.05	−3.10, −0.99	3.79	0.001	0.539\
SANS	48.2	−9.11	−11.94, −6.27	6.30	0.012	0.071
BMI	0.1	−1.01	−2.42, 0.40	1.41	0.160	0.346
Overall	51.7	−0.51	−0.67, −0.35	6.15	0.001	0.262

^a^Heterogeneity test I^2^: I^2^ < 50% means less heterogeneity, and a fixed-effects model can be chosen for merging. ^b^MD was used for combining when the scales were consistent, and Standardized Mean Difference (SMD) was used when the scales were different. ^c^*P* < 0.05 indicates a statistically significant result. ^d^*P* > 0.05 indicates no publication bias. This value was not available for less than two of the included studies.

### Adherence of AE

In this study, only four out of the seventeen included papers reported adherence data. Among these four studies, two were conducted in an inpatient setting, one in an outpatient setting, and one did not specify the setting. A Pearson’s chi-square test was performed on these four studies, revealing a marginally higher adherence rate for AE at 85.6%, compared to TAU at 84.6%. However, the difference was not statistically significant (*P* = 0.797), as detailed in Supplementary Table 1.

## Discussion

Currently, the standard approach to treating schizophrenia predominantly relies on TAU, which typically encompasses antipsychotic medication, psychiatric disorder education, medication usage guidance, and advice on daily life precautions. While timely and accurate medication administration effectively ameliorates the positive symptoms of schizophrenia, TAU has limited efficacy in improving negative symptoms, which are crucial for patients’ reintegration into normal life. Compared to adjunctive psychological interventions, adjunctive exercise-based treatments are not only effective in alleviating negative symptoms but also offer advantages in terms of easier implementation, broader applicability, and cost-effectiveness.

In the present study, we utilized exercise as a focal point, conducting a network meta-analysis to determine the most efficacious exercise modality for patients with schizophrenia. According to the SUCRA results, aerobic exercise as a standalone intervention outperformed other combinations—such as psychological interventions with AE, NAE, and TAU—in terms of appropriateness (SUCRA = 2.20%, with lower percentages indicating better effects). Interestingly, higher-intensity NAE did not yield additional benefits. These findings align with a study by Heggelund et al., which also suggested that combining aerobic and anaerobic strength-based training did not significantly reduce PANSS scores^51,52^.

Through the evaluation of key metrics such as PANSS and SANS, our study offers additional validation for the efficacy of adjunctive aerobic exercise therapy in treating schizophrenia. Specifically, we observed significant reductions in both PANSS and SANS scores. Interestingly, our findings closely align with those of Duraiswamy et al., who reported a 24% reduction in positive symptoms and an 18% reduction in negative symptoms through exercise; although the specific outcomes varied depending on the exercise modality^53^. Our results are also in agreement with Gholipour et al., who found that exercise was more effective than treatment as usual (TAU) in reducing negative symptoms, yielding a 30% decrease as measured by SANS^54^. These findings underscore the utility of adjunctive aerobic exercise interventions in alleviating negative symptoms of schizophrenia, an area where current pharmacological treatments often fall short. In contrast, our analysis on BMI revealed no significant effect of aerobic exercise, which aligns with the conclusions drawn by Firth et al.^55^, suggesting that weight management may not be an immediate outcome of exercise interventions in this population.

To enhance existing treatment protocols, our subgroup analysis offers nuanced guidance on the most effective duration and intensity of exercise regimens. Activities falling within our criteria for low-to-moderate intensity can serve as a pragmatic framework for clinicians, facilitating the development of individualized treatment strategies. We defined low-to-moderate intensity based on exercise heart rate, calculated as (220 - age) × (30%–80%), and the presence of moderate perspiration during exercise without notable post-exertional fatigue^37,38,46^. Suitable activities within this intensity range include slow walking, jogging, yoga, table tennis, Tai Chi, and general fitness exercises.

Intriguingly, our meta-analysis found no significant differences in treatment adherence between standard pharmacological treatments and adjunctive aerobic exercise (AE) interventions. We speculate that this may be attributed to multiple factors that enhance compliance in AE programs. Firstly, AE allows for both group and individual participation, which could improve patient communication, social skills, and, consequently, compliance. Secondly, several AE interventions employ strategies such as telephone follow-ups and online exercise tracking, fostering better communication between healthcare providers and patients and likely contributing to improved adherence. Lastly, the flexibility to choose the type of exercise in these interventions may promote personalization of treatment plans, thus facilitating better adherence to the regimen.

Despite these strengths, we acknowledge several limitations in this meta-analysis. The primary treatment for schizophrenia is medication-based, which varies in efficacy and side effects. Unfortunately, we were unable to account for medication-related factors as this information was not provided in the studies included in our analysis, leaving a gap in our discussion. Additionally, the number of papers included in this meta-analysis was limited, particularly with respect to SANS and BMI metrics. Future research could delve deeper into these aspects, perhaps even considering the type of medication as a focal point for further analysis. Finally, we did not conduct a systematic review of literature related to psychological interventions for schizophrenia patients in this analysis. Consequently, we were unable to compare the effects of psychological interventions to those of exercise-based interventions as non-pharmacological treatment options. This will be a focus for further investigation.

## Conclusion

Combining conventional treatment with aerobic exercise interventions proves more effective in alleviating symptoms of schizophrenia than conventional treatment alone. This approach is particularly beneficial in ameliorating negative symptoms and shows higher adherence rates among patients. The type of aerobic exercise can be tailored to the individual’s preferences, with low-to-moderate intensity options such as yoga, slow walking, jogging, gymnastics, and table tennis being recommended. The optimal duration for the intervention is between two and three months, with an advisable frequency of 3–5 exercise sessions per week, each lasting 35–45 min.

### Supplementary information

Supplementary Fig.1, Supplementary Fig.2, Supplementary Fig.3, Supplementary Table 1

## Data Availability

All data are available upon reasonable request.
