# Supplementary material for: Efficacy and feasibility of aerobic exercise interventions as an adjunctive treatment for patients with schizophrenia: a meta-Analysis
Source: Schizophrenia (Heidelb). 2024 Jan 2;10(1):2. doi: 10.1038/s41537-023-00426-0 (PMC10851701; doi:10.1038/s41537-023-00426-0)
Supplement: Supplementary file 1 — Supplementary Fig.1, Supplementary Fig.2, Supplementary Fig.3, Supplementary Table 1 [file 41537_2023_426_MOESM1_ESM.docx]

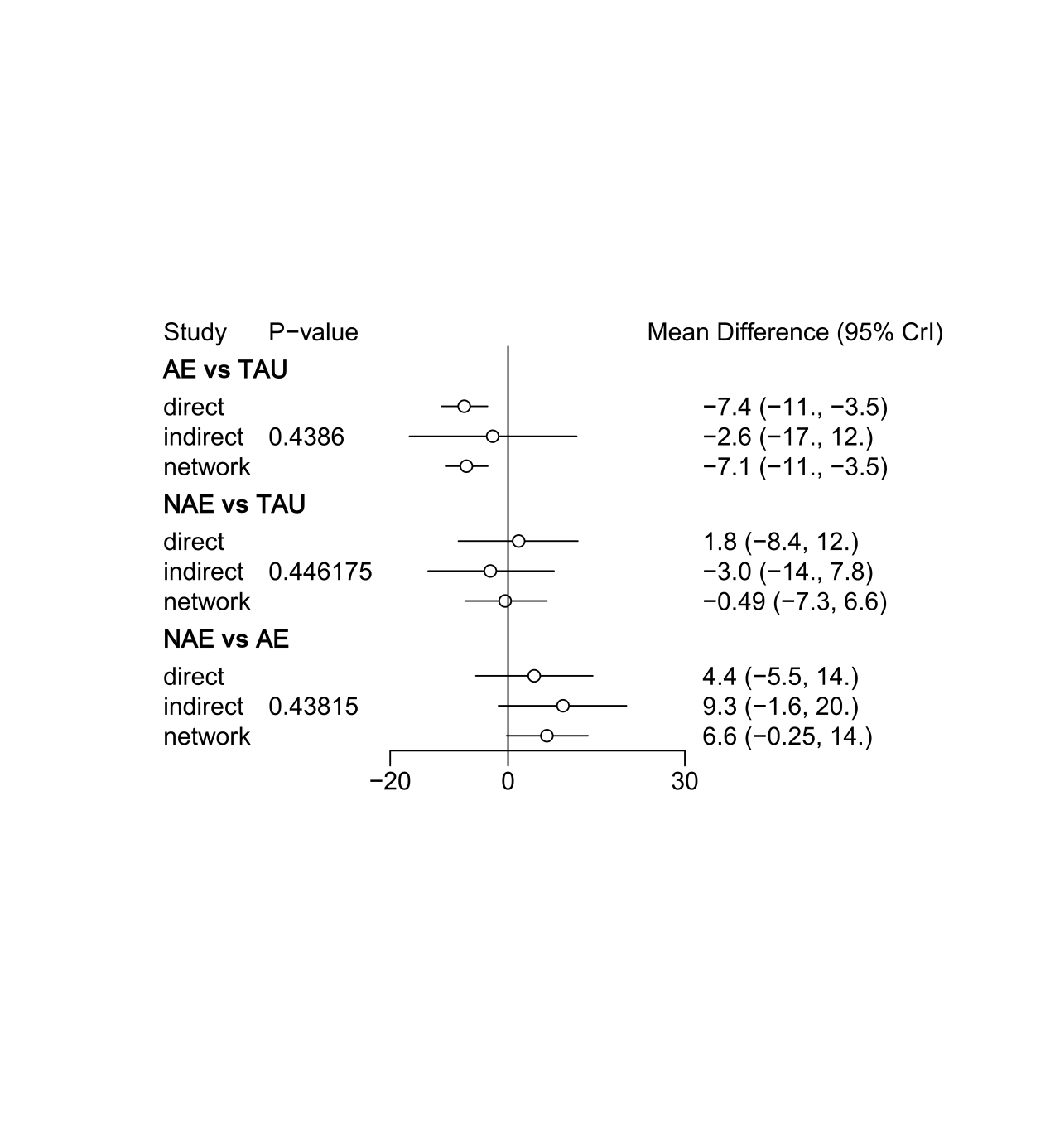


**Supplementary Fig.1 Node cutting forest figure.** *P*-value >0.05 indicates that the inconsistency test result is not significant.


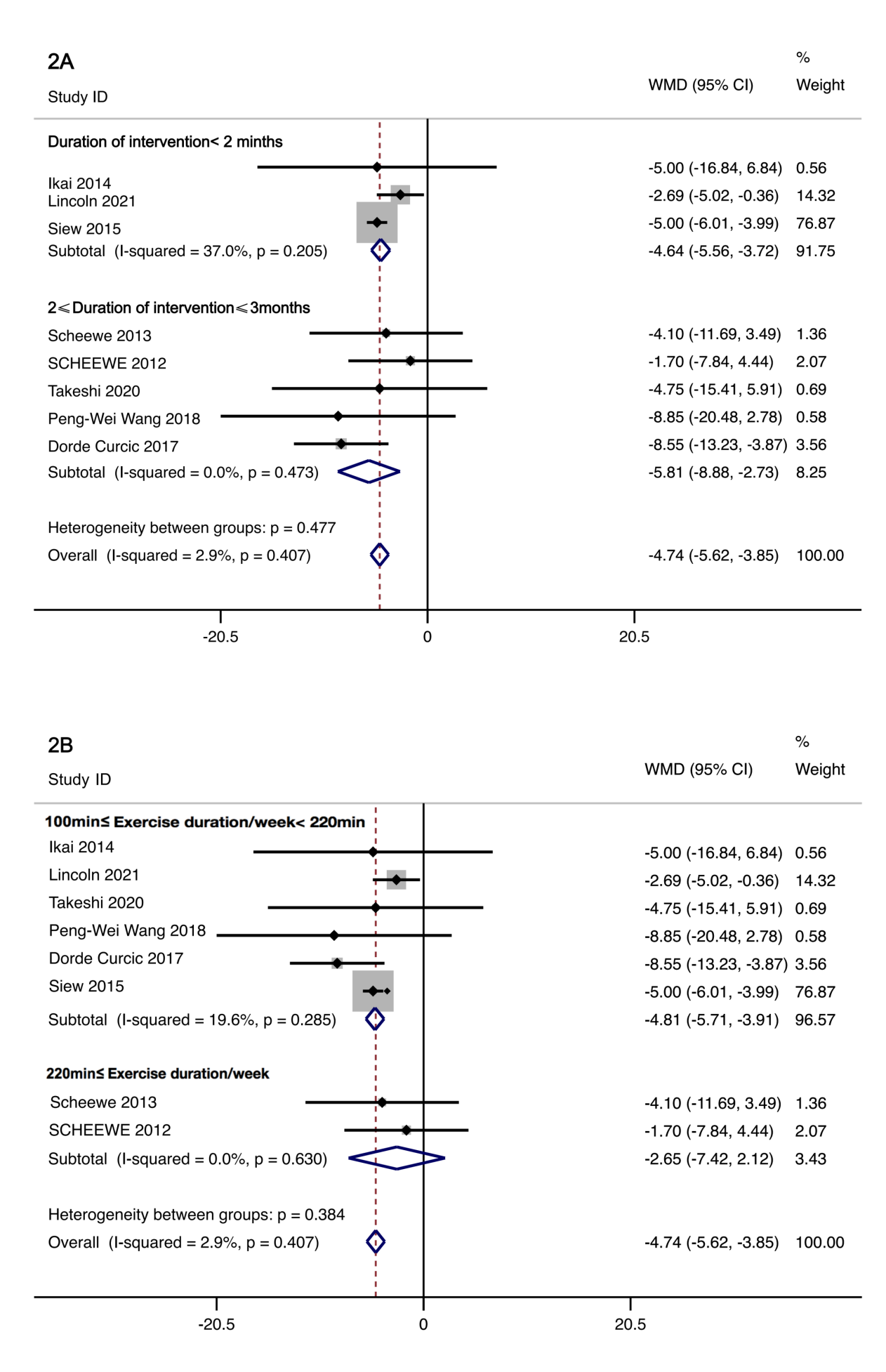


**Supplementary Fig.2 Subgroup forest plots -PANSS.** 2A: Subgroup forest plots for duration of intervention. 2B: subgroup forest plots for total duration of exercise per week.





**Supplementary Fig.3 Forest plot for the PANSS sub-scales.**

**Supplementary Table 1. Adherence of AE.**

| **Group** | **No. of people** | **Adherence (n,%)** | **Non-adherence (n,%)** | **Person Chi-Square** | **P** |
| --- | --- | --- | --- | --- | --- |
| AE | 153 | 131(85.6%) | 22(14.4%) | 0.67 | 0.796 |
| TAU | 149 | 126(84.6%) | 23(15.4%) |  |  |
